# Structures and mechanisms of the RNA m ^6^A writer

**DOI:** 10.3724/abbs.2024152

**Published:** 2024-09-04

**Authors:** Ting Deng, Jinbiao Ma

**Affiliations:** State Key Laboratory of Genetic Engineering Collaborative Innovation Centre of Genetics and Development Department of Biochemistry and Biophysics Institute of Plant Biology School of Life Sciences Fudan University Shanghai 200438 China

## Abstract

*N* ^6^-methyladenosine (m ^6^A) is the most prevalent epigenetic modification found in eukaryotic mRNAs and plays a crucial role in regulating gene expression by influencing numerous aspects of mRNA metabolism. The m ^6^A writer for mRNAs and long non-coding RNAs consists of the catalytic subunit m ^6^A-METTL complex (MTC) (including METTL3/METTL14) and the regulatory subunit m ^6^A-METTL-associated complex (MACOM) (including HAKAI, WTAP, VIRMA, ZC3H13, and RBM15/15B). In this review, we focus on recent advances in our understanding of the structural and functional properties of m ^6^A writers and the possible mechanism by which they recognize RNA substrates and perform selective m ^6^A modifications.

## Introduction

More than one hundred chemical modifications have been detected on various RNAs. Among them, transfer RNA (tRNA) has been found to be the most modified, followed by ribosomal RNA (rRNA) [1]. mRNA also contains a variety of chemical modifications, including *N* ^7^-methylguanosine (m ^7^G), *N* ^6^-methyladenine (m ^6^A), 5-methylcytosine (m ^5^C), *N* ^1^-methyladenine (m ^1^A), pseudouracil (pseudouridine, Ψ), and inosine (I) [ 1, 2]. Among these modifications, m ^6^A is the most abundant modification of eukaryotic messenger RNA (mRNA), and m ^6^A modification also occurs in a variety of bacteria and RNA viruses [3].

The discovery of m ^6^A modifications can be traced back to the 1970s. Owing to the limitations of m ^6^A modification-related methods, research related to m ^6^A modification of RNA has not progressed [4]. With the development of technology, researchers have developed a series of methods, including immunoprecipitation, mass spectrometry analysis, and antibody identification [ 5‒ 12], to explore the distribution of m ^6^A modifications on RNA and its biological functions. These technologies advance m ^6^A research by allowing researchers to detect and quantify m ^6^A modifications more accurately. m ^6^A modifications are abundant in the 3′ UTRs and long exons of eukaryotic mRNAs and play crucial roles in many physiological and pathological processes [ 5, 13] .

In eukaryotes, m ^6^A modifications of RNA are catalyzed by three main classes of SAM-dependent MTases: the first class comprises METTL3/METTL14 and METTL4, the second class comprises METTL16, and the last class comprises METTL5/TRMT112 and ZCCHC4. Among them, METTL5/TRMT112 and ZCCHC4 are two ribosomal RNA (rRNA) MTases [ 14‒ 21]. METTL4 belongs to the MT-A70 family, which is similar to METTL3/METTL14, and specifically catalyzes *N* ^6^-methylation on A30 or A31 with 2′- *O*-methyl modification in U2 small nuclear RNA (snRNA) [ 22‒ 25]. METTL16 specifically catalyzes m ^6^A modification of U6 snRNA and MAT2A mRNA on the consensus sequence of UACAGARAA (modified A underlined) [ 1, 26‒ 38]. METTL3/METTL14 form heterodimers and play major roles in m ^6^A modification of mRNAs and non-coding RNAs [ 39‒ 43].

m ^6^A modification is a reversible chemical modification that is added by m ^6^A MTase [ 44, 45] and removed by m ^6^A demethylase [ 46, 47], and m ^6^A reader proteins recognize m ^6^A modifications [ 48, 49]. m ^6^A reader proteins bind to RNA containing m ^6^A modifications and are responsible for regulating RNA metabolism, including the processing of mRNA precursors [50], mRNA translation efficiency [51], transcription stability [48], and RNA transport [52].

In this review, we focus on recent progress in the structures, functions and regulation of m ^6^A methyltransferases (MTases) for mRNAs and noncoding RNAs, especially the mechanism by which m ^6^A methyltransferases (MTs) recognize RNA substrates and selectively modify them.

## RNA m ^6^A Writers

**Figure 1:**
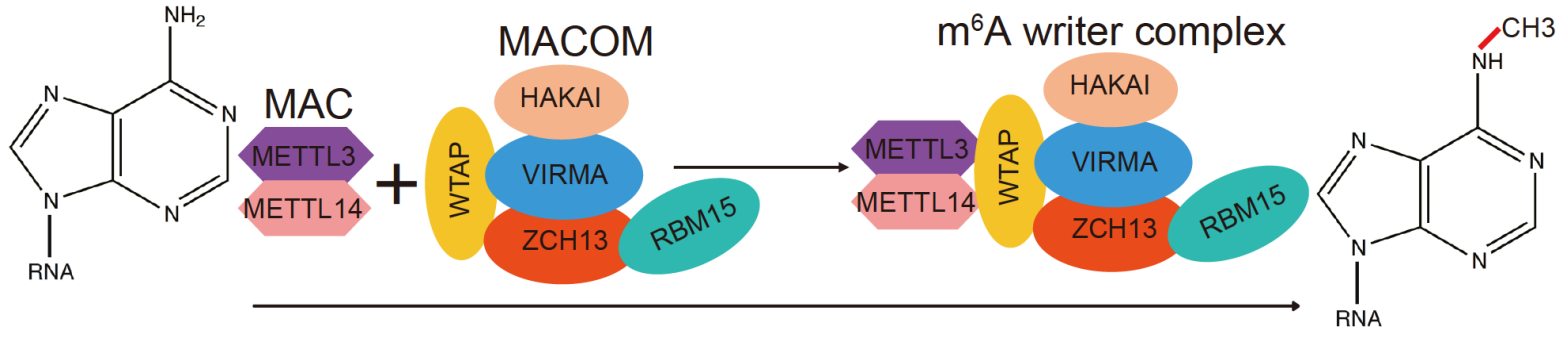
Figure 1 Model of RNA m ^6^A methylation by m ^6^A writers composed of MACs and MACOM

The RNA m ^6^A writer was first discovered by Bokar *et al*. [53]. These authors also separated the components of the enzyme through chromatography *in vitro*, which are mainly divided into three parts with molecular weights of 30 kDa, 200 kDa and 875 kDa: MT-A1, MT-A2 and MT-B, respectively [53]. Through *in vitro* MTase experiments, these authors reported that these three components are necessary for m ^6^A MTase activity *in vitro* [53]. However, subsequent studies revealed that only MT-A2 and MT-B are required for m ^6^A MTase activity. Through a cross-linking separation experiment of S-adenosylmethionine (SAM), a 70 kDa protein capable of binding SAM was identified in the MT-A2 component, named MT-A70, also known as METTL3 [54]. A later study reported other MTases in mammals, METTL14 and WTAP [44], and these three proteins, METTL3/METTL14/WTAP, form the core of the MTase complex. In addition to this complex, other proteins (HAKAI, VIRMA, ZC3H13, RBM15, *etc*.) called the m ^6^A METTL-associated complex (MACOM) also play roles in m ^6^A modification processes, such as substrate binding, catalytic efficiency, stability, and substrate selectivity [ 55‒ 59]. The m ^6^A MTase complex is approximately 1000 kDa in size and includes MTase-like 3 and 14 (METTL3/METTL14), Wilms’ tumor 1-associated protein (WTAP), KIAA1429 (VIRMA), zinc finger CCCH domain-containing protein 13 (ZC3H13), RNA binding motif protein 15/15 paralogue (RBM15/RBM15B), and the E3 ubiquitin ligase CBLL1 (HAKAI) [ 45, 56‒ 60]. Among them, the heterodimer formed by METTL3 and METTL14 constitutes a catalytic subunit of the m ^6^A MTase [44], also called the m ^6^A-METTL complex (MAC) [56]. Structural and biochemical studies have shown that METTL3 binds to the cofactor SAM and has catalytic activity, whereas METTL14 can stabilize the conformation of METTL3 and promote substrate binding [ 39‒ 41, 61]. In addition, MACOM was found to play regulatory roles in m ^6^A MTase activity and m ^6^A modification site selectivity in different species [ 45, 56‒ 58, 60] ( Figure 1). MACOM is conserved in most eukaryotes, such as *Drosophila*, mice, humans, and plants [ 56, 62]. Protein‒protein interaction analysis [63] revealed that the four proteins HAKAI, WTAP, VIRMA and ZC3H13 in MACOM can form functional complexes in human cells [ 56, 58, 59].

### METTL3/METTL14

**Figure 2:**
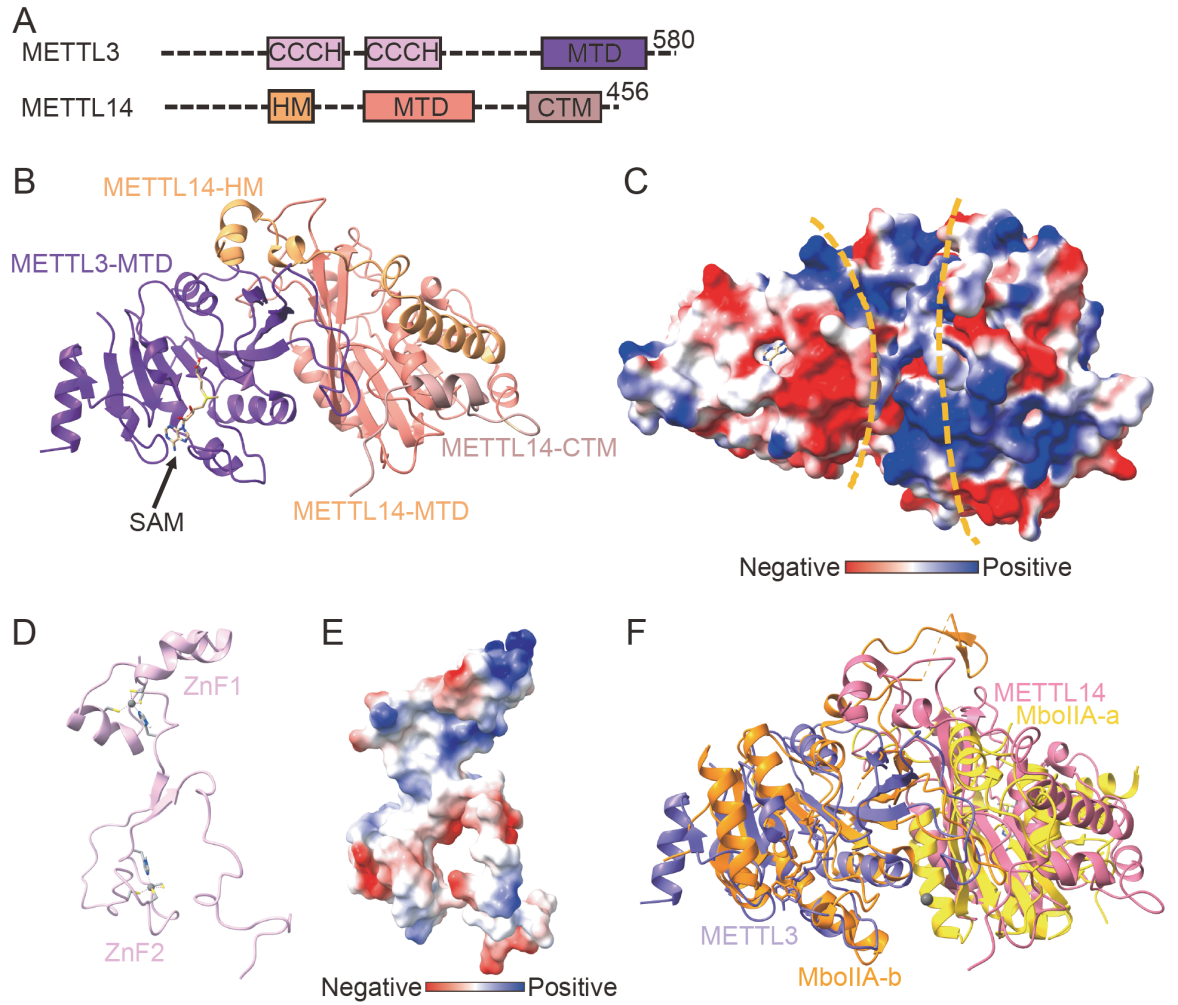
Figure 2 Domain organization and structures of the MAC (A) Domain organization of METTL3/METTL14. (B) The overall structure of the heterodimer complex of the MTase domain of METTL3/METTL14 (MTD3/MTD14), and the PDB ID is 5IL1. (C) Surface representation of METTL3/14, the surface is colored according to electrostatic potential, and the PDB ID is 5IL1. (D) The structure of the ZnF domain of METTL3, and the PDB ID is 5YZ9. (E) Surface representation of the ZnF domain of METTL3, the surface is colored by electrostatic potential, and the PDB ID is 5YZ9. (F) Structural alignment of METTL3/METTL14 and MboIIA.

METTL3 was originally called MT-A70 and was later called METTL3; METTL3 belongs to the RNA MTase family. Evolutionary analysis revealed that METTL14 and METTL3 belong to the MT-A70 family, and they share 43% sequence similarity, indicating that METTL14 may be a redundant protein of METTL3 or may have its own m ^6^A modification pattern [ 44, 64 ]. Human METTL3 and METTL14 form a complex at a 1:1 ratio. The N-terminus of METTL3 contains an N-terminal extension (N-extension), a leader helix (LH) and a nuclear localization signal (NLS) [18], followed by two consecutive CCCH zinc finger domains (ZF1 and ZF2) and an MTase domain (MTD) at the C-terminus [18]. The N-terminus of human METTL14 has an N-terminal extension (N-extension), followed by an MTase domain and an unstructured, disordered C-terminal extension (C-extension) [18] ( Figure 2A).

In 2016, three research groups solved the crystal structure of the catalytic domain of METTL3/METTL14 heterodimers [ 39, 40, 61 ]. The core of each MTase subunit is a Rossman fold domain comprising eight-stranded β-sheets flanked by four α-helices. The MTase domains of METTL3/METTL14 form heterodimers through hydrogen bonds and hydrophobic interactions ( Figure 2B,C) [40]. Many amino acids at the interface of the dimer are highly conserved, and these interactions stabilize the structures of METTL3/METTL14 [ 39, 40]. The MTase domains of METTL3/METTL14 are highly similar to domains containing the Rossman fold of other MTases, such as the bacterial adenine-specific DNA MTases MboIIa and RsrI ( Figure 2F) [ 65, 66]. The pattern of the METTL3/METTL14 heterodimer complex is similar to that of *N* ^6^-DNA MTases in prokaryotes, such as EcoP15I [67]. However, its dimer structure differs from the dimerization mode of other multisubunit RNA MTases, such as Bud23-Trm12 or Trm9-Trm112 [ 68, 69]. In the crystal structure of METTL3/METTL14, there is an AdoMet binding pocket in METTL3 but not in METTL14 ( Figure 2B). Structural analysis revealed that there are many cofactor-binding amino acids in METTL3, including the catalytic loop DPPW (D395–W398) motif, of which D395 is required for SAM binding and MTase activity, and W398 interacts with methylated adenine through π-π stacking during the process of binding substrates. The DPPW in METTL3 is replaced by an EPPL motif (E192–L195) in METTL14, which lacks key amino acids, which makes METTL14 unable to bind to SAM and has no catalytic activity [39]. Taken together, these results indicate that although METTL14 lacks cofactor-binding ability and catalytic activity, it can increase the affinity of SAM for MACs [70]. When METTL14 is present, the MTase activity of METTL3 is greatly enhanced [40]. In summary, only METTL3 has catalytic activity, and METTL14 has no catalytic activity; however, METTL14 can promote the binding ability of METTL3 to SAM and increase the m ^6^A MTase activity of METTL3 through allosteric regulation. In 2018, a research group analyzed the nuclear magnetic structure of the zinc finger domain of METTL3. Two zinc finger domains of METTL3, ZnF1 and ZnF2, are connected by a β-sheet ( Figure 2D,E) [41]. The zinc finger domain is crucial for the MTase activity of METTL3/METTL14 because the zinc finger domain of METTL3 helps recognize the DRACH motif [ 41, 44]. However, the affinity of the zinc finger domain for the DRACH motif is relatively low, which allows METTL3 to quickly dissociate from the RNA substrate after completing methylated modification, thereby improving the catalytic effect of METTL3 and increasing the abundance of m ^6^A modifications on mRNAs [ 41, 44]. In 2023, a research group analyzed the crystal complex structure of the catalytic domain of METTL3/METTL14 with the reaction product *N* ^6^-methyladenosine monophosphate and reported that the binding pocket of the reaction product is very conserved and is approximately 16 Å from the SAM binding pocket [42]. They proposed a model in which A is methylated into m ^6^A and then rotated, changing the state of METTL3/METTL14 so that it can recognize m ^6^A [42]. Previous studies have shown that cancer-related METTL3/METTL14 mutations cannot distinguish methylated and unmethylated adenine, which leads to changes in RNA and m ^6^A binding, leading to changes in m ^6^A modification levels in cells and disease [42].

Previous studies have shown that the methylation of METTL3/METTL14 is sequence specific, but it has no structural preference for RNA substrates [44]. Biochemical studies have shown that the zinc finger domain and catalytic domain of METTL3, the catalytic domain and the C-terminal extension region of METTL14 interact with RNA substrates, but the molecular mechanism by which METTL3/METTL14 specifically recognizes the DRACH sequence on the RNA substrate is unclear [ 39‒ 41]. The molecular mechanism by which METTL3/METTL14 bind to RNA substrates remains to be revealed.

### MACOM

**Figure 3:**
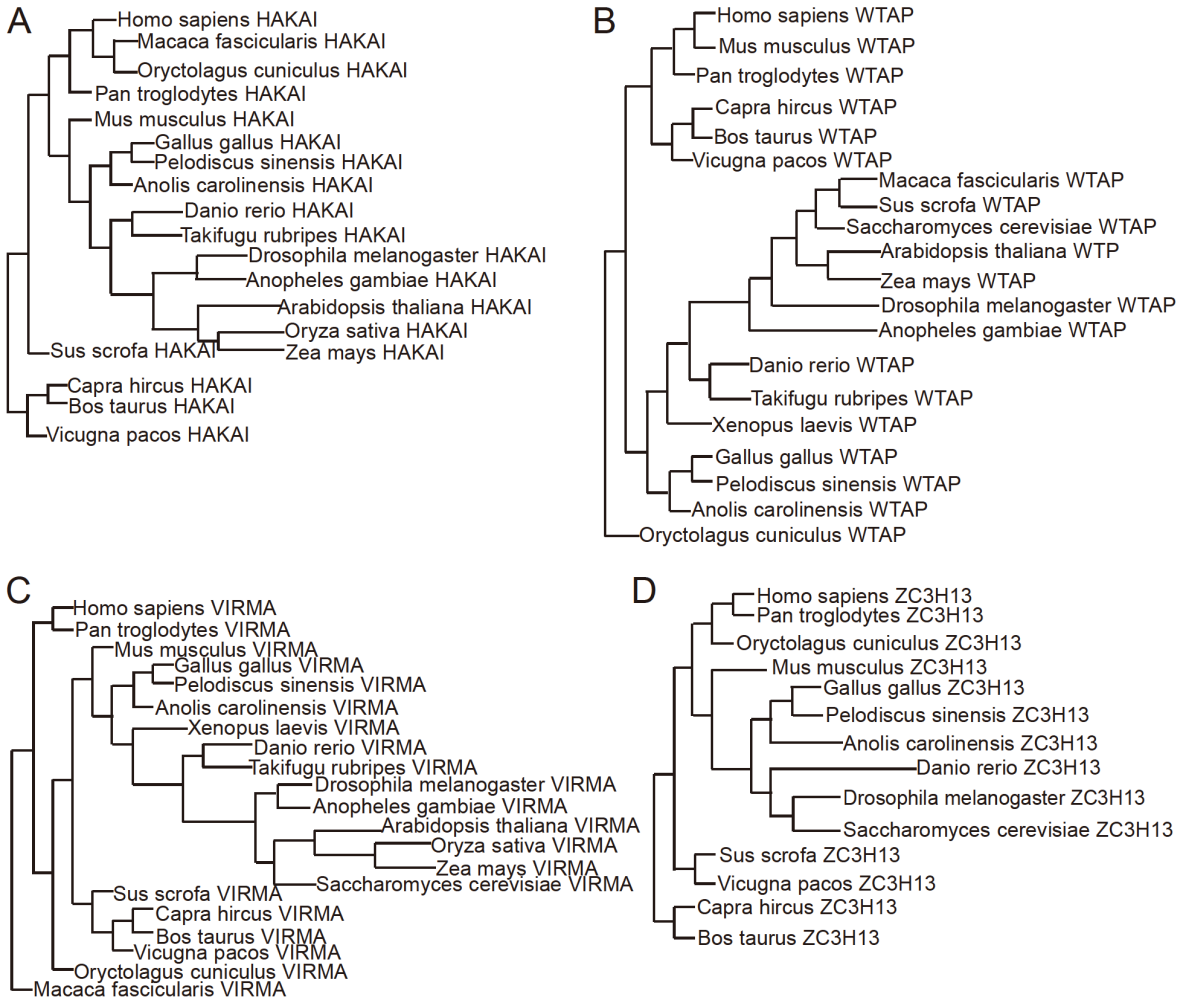
Figure 3 Phylogenetic tree of HAKAI, WTAP, VIRMA, and ZC3H13 (A) Phylogenetic tree of HAKAI. (B) Phylogenetic tree of WTAP. (C) Phylogenetic tree of VIRMA. (D) Phylogenetic tree of ZC3H13.

In addition to the catalytic core components of MAC, more components of MACOM have been identified in higher animals and plants, suggesting that more regulation is required for m ^6^A modification in complicated biological systems [71]. Although the components of MACOM are not widely present, WTAP coevolves with the METTL3/METTL14 complex in most eukaryotic species [71]. HAKAI, WTAP, VIRMA, and ZC3H13 coexist in metazoans [63]. In plants, only HAKAI, WTAP, and VIRMA are copurified in multicellular plants, whereas ZC3H13 has not been identified by biochemical purification or protein sequence BLAST [72]. Evolutionary analysis revealed that HAKAI, WTAP, and VIRMA all exist in metazoans and higher plants and are conserved. ZC3H13 also exists in metazoans and is relatively conserved ( Figure 3). Previous structural and biochemical studies have shown that ZC3H13 mainly interacts with other proteins in MACOM through its C-terminus [ 73, 58]. Many previous studies have shown that multiple components of MACOM play very important roles in the m ^6^A modification process. WTAP recruits METTL3/METTL14 to nuclear speckles and is crucial for unique localization [ 44, 60, 74 ]. Furthermore, RBM15 can bind to WTAP and recruit the MTC to specific RNA sites for m ^6^A modification [75]. ZC3H13 interacts with WTAP to retain the MTC in nuclear speckles via its C-terminus and thereby promotes its function [ 56, 73, 58 ].

#### HAKAI

**Figure 4:**
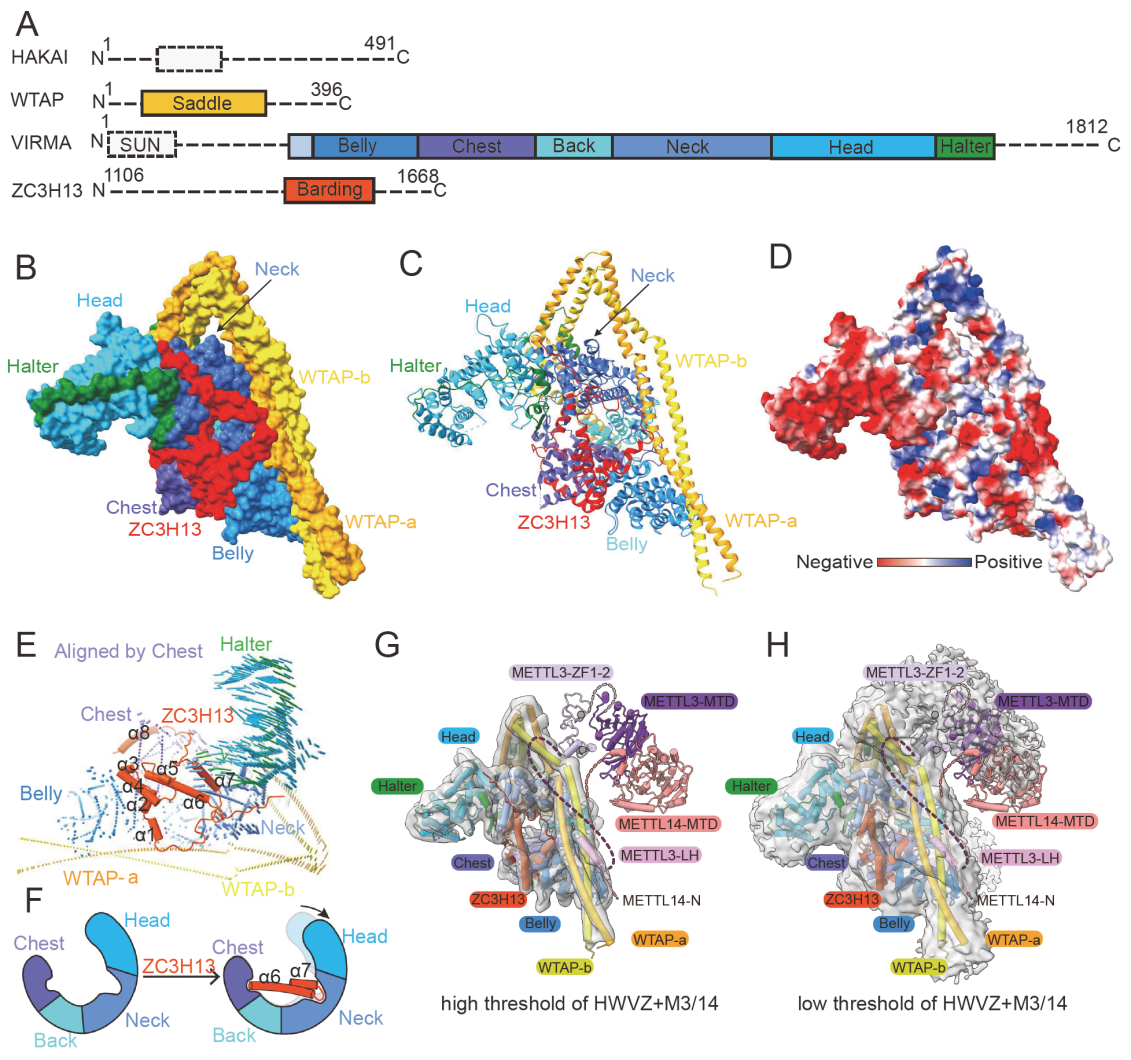
Figure 4 Domain organization and structures of MACOM (A) Domain organization of the HWVZ. (B) Surface representation of HWVZ, and the PDB ID is 7VF2. (C) Cartoon representation of HWVZ, and the PDB ID is 7VF2. (D) Surface representation of the MACOM and the surface is colored according to the electrostatic potential. PDB ID is 7VF2. (E) Conformational changes in the HWV complex upon ZC3H13, aligned by the Chest domain of VIRMA. (F) Model of ZC3H13 α6 and α7 binding-induced conformational changes in HWV. (G) Model of the m6A writer complex in the apo state on the basis of the HWVZ+METTL3/14 cryo-EM map and biochemistry data, the cryo-EM map shown at a high threshold level (level = 0.1). The light-coral dashed line represents the N-terminus of METTL14, purple and lavender dashed lines represent the N-terminus and the linker between the zinc finger domain and MTA domain of METTL3, respectively. The EMD accession ID is EMD-34169, and the PDB IDs are 7VF2, 5IL1, 5YZ9. (H) Model of the m6A writer complex in the apo state based on HWVZ + METTL3/14 cryo-EM map and biochemistry data; the cryo-EM map is shown at low threshold level (level = 0.013). The light-coral dashed line represents the N-terminus of METTL14, purple and lavender dash lines represent the N-terminus and the linker between the zinc finger and MTA domain of METTL3, respectively. The EMD accession ID is EMD-34169, and the PDB IDs are 7VF2, 5IL1, and 5YZ9.

HAKAI is a potential E3 ubiquitin ligase with a complete C3HC4 RING domain and C2H2 domain ( Figure 4A). HAKAI was initially identified as an E-cadherin-binding protein that degrades E-cadherin by ubiquitinating E-cadherin, and functional studies revealed that HAKAI plays roles in cell proliferation and tumor progression *in vivo* [76]. In *Drosophila*, deletion of HAKAI results in a decrease rather than an increase in other components of the m ^6^A MTase, indicating that HAKAI may not function as an E3 ubiquitin ligase in the m ^6^A MTase complex [77].

In plants, *Drosophila*, and mammals, HAKAI colocalizes with other components of the m ^6^A MTase. HAKAI is a core component of the MTase complex in *Drosophila* and humans. In *Drosophila*, knocking down *HAKAI* results in reduced m ^6^A modification levels and affects m ^6^A modification-related functions, including sex determination [72]. Although HAKAI is a core component of the m ^6^A MTase complex, *HAKAI* knockdown has a weaker effect on m ^6^A modification levels than other components in MACOM [72]. Studies have shown that the HYB (RING+pTyr-B) domain of HAKAI forms a dimer [ 78, 79]. Other studies have demonstrated through coimmunoprecipitation experiments that HAKAI interacts with the N-terminal SUN domain of VIRMA in *Drosophila* and humans and that knocking down *HAKAI* decreases the stability of several other subunits in the MTase complex *in vivo* [ 77, 80]. These findings indicate that HAKAI may stabilize the structure of the m ^6^A MTase by interacting with other components of the m ^6^A MTase *in vivo*, thereby participating in the regulation of m ^6^A modification. However, there is currently no structure of m ^6^A MTases containing HAKAI. Therefore, the molecular mechanism of HAKAI in m ^6^A modification should be further explored in the future.

#### WTAP

WTAP was originally named because of its association with the tumor suppressor gene Wilms tumor 1 (WT1), mutations in which cause Wilms tumor, a rare form of kidney cancer that occurs almost exclusively in children [81]. Wilms’ tumor 1-associating protein (WTAP) is also an important component of the m ^6^A MTase complex, which is required for the catalytic activity of METTL3/METTL14 *in vivo*. WTAP was originally discovered in plants to play a role in m ^6^A modification [82]. WTAP is highly conserved in higher eukaryotes. FIP37 is a homologous protein of WTAP in *Arabidopsis*. It strongly interacts with MTA, indicating that this protein may be involved in the m ^6^A modification process [82]. In 2012, Agarwala *et al*. [83] reported that Mum2, a homologous protein of WTAP in *Saccharomyces cerevisiae*, is involved in m ^6^A modification.

WTAP consists of an N-terminal region, a coiled-coil domain and a C-terminal region [84]. Several studies have demonstrated through Co-IP experiments that WTAP can interact with the METTL3/METTL14 heterodimer, anchoring METTL3/METTL14 to RNA [84]. In 2022, two research groups analyzed m ^6^A MTase structures containing WTAP [ 73, 85]. Among them, WTAP forms a saddle-shaped homodimer through coiled-coil interactions, including four tandem helices (H1-H4/H1′-H4′), three connecting regions (L1-L3/L1′-L3′) and three unique corners. The formation of the dimer relies on strong interactions between two monomers, where the interface area between two WTAP monomers is approximately 4620 Å ^2^. However, the two monomers of WTAP have a lower cross-correlation coefficient and lower R.m.s. deviations (15.9 Å), indicating that the conformations of the two monomers are not the same. Biochemical and structural results revealed that WTAP mainly interacts with the leader helix at the N-terminus of METTL3 and the N-terminus and C-terminus of METTL14 through its H3-H4/H3′-H4′ ( Figure 4 G,H) [73]. These findings indicate that WTAP may indirectly participate in the regulation of m ^6^A modification by stabilizing the structure of METTL3/METTL14 or directly participate in the regulation of m ^6^A modification by recruiting other factors [ 44, 83 , 86]. However, the molecular mechanism of WTAP in m ^6^A modification is still unclear and should be further explored in the future.

#### VIRMA

VIRMA (Vir-like m ^6^A MTase), another important component of the m ^6^A MTase, is a homologue of *Drosophila* Virilizer protein in mammals [59]. Mass spectrometry revealed that VIRMA is a protein that interacts with WTAP [62]. Subsequent proteomic studies revealed similar interactions [45]. Virilizers interact with WTAPs, are involved in the splicing of pre-mRNAs, and are associated with sex determination in *Drosophila* [45]. Knocking out *Virilizer* also results in a dramatic reduction in m ^6^A modification, indicating that *Virilizer* is necessary for m ^6^A modification [45]. Other studies have reported that most VIRMA-dependent m ^6^A modifications are located in the 3′UTR and near the stop codon of mRNA and that m ^6^A modification sites are close to polyadenylation sites in the 3′UTR. Knocking down *VIRMA* leads to elongation of the 3′UTR, suggesting that the m ^6^A MTase plays a role in regulating polyadenylation [59].

The human VIRMA protein contains 1812 amino acids and a SUN domain at the N-terminus [59]. Two research groups analyzed the cryoelectron microscopic structure of the MACOM complex containing VIRMA. VIRMA contains twenty ARM-like (ARML) structures, forming a horse-like structure [ 73, 85 ]. Therefore, each domain of VIRMA is named the Belly domain, Chest domain, Back domain, Neck domain, or Head domain. The Belly domain and Chest domain are connected by Connect Helix-1, and the Chest domain and Back domain are connected by a similar Connect Helix-2. There is a long loop structure behind ARML20, which interacts with L1/L1′ in the WTAP homodimer, similar to a horse’s Halter, so it is called the Halter domain. Although the overall surface charge of VIRMA is mostly negative, several small regions are positively charged ( Figure 4F). These regions are also relatively conserved ( Figure 4G), suggesting that they may be important for interactions with other components of MACOM, especially WTAP. The interface area between VIRMA and WTAP-a is approximately 2778 Å ^2^, including three salt bridges, fifteen hydrogen bonds, and many nonbonded contacts. The interface area between VIRMA and WTAP-b is approximately 3303 Å ^2^ . There are two positively charged regions on the Back and Belly domains of VIRMA, which contain highly conserved amino acids. These parts are H1/H1′ and H4/H4′ of the WTAP, respectively. Many amino acids in these regions of VIRMA interact with WTAP, including several hydrogen bonds. These interactions make WTAP and VIRMA form the core structure of MACOM [73]. In addition, biochemical experiments have shown that the C-terminus of VIRMA is important for binding RNA substrates *in vivo* [73].

#### ZC3H13

Another known m ^6^A MTase-related protein is ZC3H13 (zinc finger CCCH-type containing 13) [ 55, 56 , 58]. ZC3H13 is localized in the nucleus in mammals. Studies have reported that ZC3H13 regulates the localization of m ^6^A MTase to the nucleus by interacting with RBM15 *in vivo*, and this interaction may be a direct protein-protein interaction rather than an RNA-dependent interaction [56]. In addition, several studies have conducted Co-IP experiments on ZC3H13 truncation mutants and HAKAI/WTAP/VIRMA strains to explore which region of ZC3H13 interacts with these three proteins. ZC3H13 mainly interacts with the other three proteins through its C-terminal 1461–1729 in mice [58]. This study also revealed that knocking down *ZC3H13* significantly affects m ^6^A modification levels on RNA and the proportion of HAKAI/WTAP/VIRMA that enter the nucleus *in vivo* [58]. Moreover, after being transferred to ZC3H13, the localization of HAKAI/WTAP/VIRMA in the nucleus returns to normal, suggesting that ZC3H13 can mediate m ^6^A MTase entry into the nucleus directly or indirectly by interacting with other proteins [58]. ZC3H13 controls the pluripotency of embryonic stem cells by regulating m ^6^A modification [58]. Knuckles *et al* . [58] reported that ZC3H13 in mice and Flacc (the homologous protein of ZC3H13 in *Drosophila*) mediate the interaction between RBM15 and WTAP. Flacc also regulates alternative splicing of *Drosophila*’s Sxl in a m ^6^A-dependent manner, thereby affecting *Drosophila*’s sex determination and dosage compensation [58].

ZC3H13 is a zinc finger protein that contains a zinc finger domain at its N-terminus, and approximately 80% of the other sequences are of low complexity. In 2022, a group analyzed the structure of the MACOM complex containing the C-terminal structure of ZC3H13 [73]. ZC3H13 is different from the rigid conformations of WTAP and VIRMA, which form saddle shapes and horse shapes, respectively, and the structure of ZC3H13 is relatively flexible. The C-terminus of ZC3H13, which is one-tenth of the full length of ZC3H13 and contains eight helices, can be observed in the cryo-EM structure. The interaction between the Barding domain of ZC3H13 and VIRMA is strong, and the interface area between the two proteins is approximately 4435 Å ^2^ ( Figure 4B,C). It contains seven salt bridges and nineteen hydrogen bonds. However, the interaction between the boarding domain of ZC3H13 and WTAP is relatively weak, and the interface area is only approximately 256 Å ^2^. ZC3H13 interacts with the Belly, Chest and Neck domains of VIRMA, which is similar to a barding that protects the body of the horse ( Figure 4E,F). Therefore, this part of ZC3H13 is called the boarding domain. Moreover, sequence alignment revealed that this part of ZC3H13 is relatively conserved. α1-5 in the Barding domain of ZC3H13 interacts with the Belly domain of VIRMA, α8 interacts with the Chest domain, and α6 and α7 interact with the Neck domain. Therefore, the Neck domain of VIRMA is pulled away from the Belly and Chest domains, spreading apart the conformation of VIRMA at approximately 6 Å. Biochemical experiments have shown that the C-terminal region of ZC3H13 has a great impact on RNA binding and m ^6^A MTase activity *in vitro*, indicating that ZC3H13 may be involved in stabilizing the structure of the MAC-MACOM complex or directly participating in binding RNA substrates.

#### RBM15

RBM15 and the *Drosophila* homologous protein Nito were first discovered to interact with WTAP. This interaction can regulate the alternative splicing of *Drosophila’s* Sxl [87]. Knocking down *RBM15* significantly affects m ^6^A modification levels in cells and results in embryonic lethality in *Drosophila* and mice [ 57 , 88]. iCLIP experiments revealed that RBM15/RBM15B bind to U-rich regions on mRNAs close to m ^6^A modification sites [57], suggesting that RBM15/RBM15B bind to U-rich regions, possibly by recruiting m ^6^A MTases to methylate DRACH motifs near U-rich regions [57]. However, not all DRACH motifs are located near U-rich regions, indicating that other factors may be involved in regulating m ^6^A modification site selectivity. RBM15 is expressed in many eukaryotes and belongs to a large family of RNA-binding proteins. Different RNA-binding proteins can target specific mRNAs under different conditions and may replace the function of RBM15 [89].

**Figure 5:**
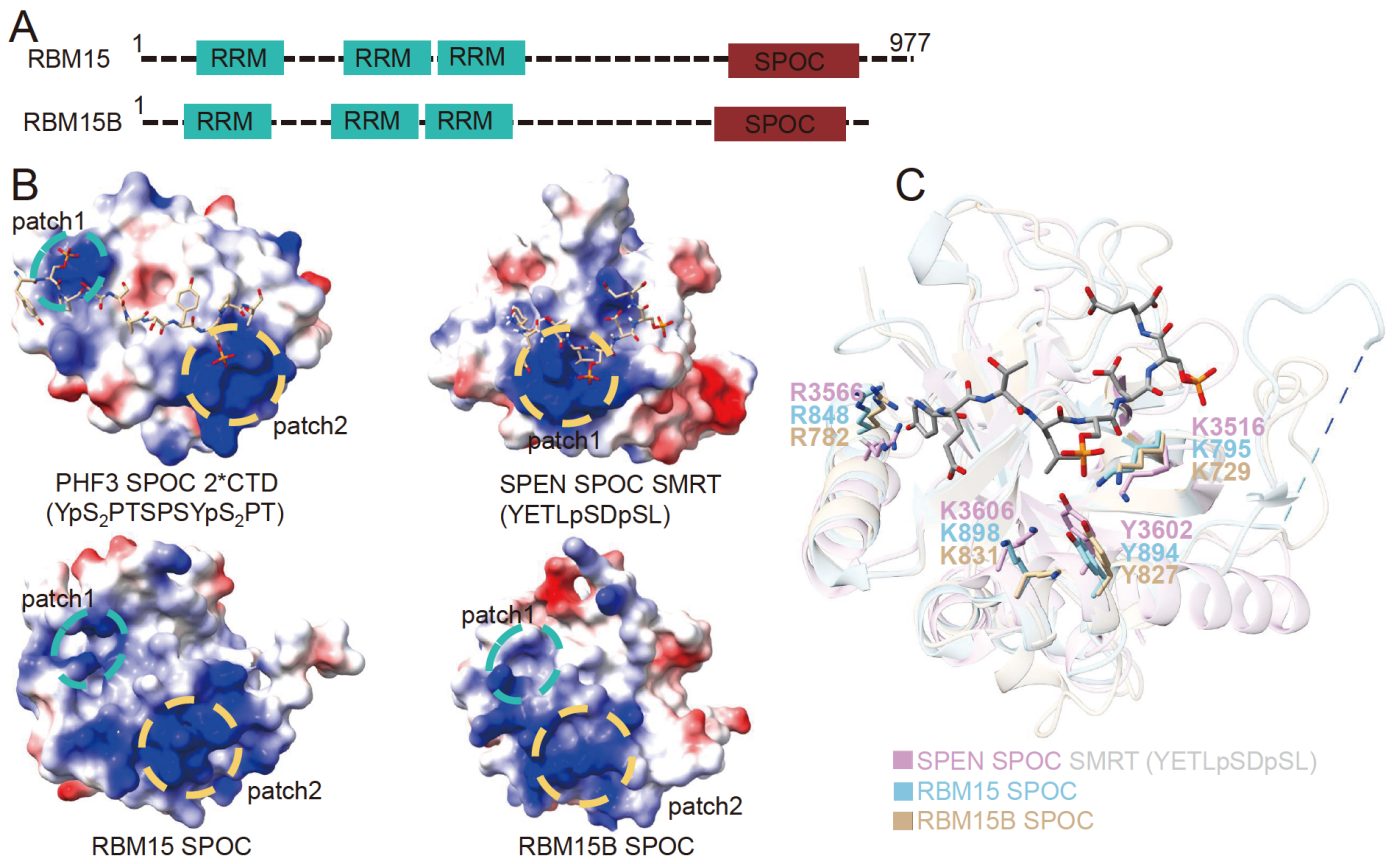
Figure 5 Domain composition and structures of RBM15/RBM15B (A) Domain organization of RBM15/RBM15B. (B) Surface representation of the SPOC domain of PHF3, SPEN, RBM15, and RBM15B, the surface is colored according to the electrostatic potential. The upper left corner is the complex structure of the SPOC domain of PHF3 with 2*CTD, in which the SPOC is displayed as a surface, and 2*CTD is displayed as a stick; the PDB ID is 6IC8. The upper right corner is the complex structure of the SPOC domain of SPEN with SMRT, in which the SPOC is displayed as a surface, SMRT is displayed as a stick, and the PDB ID is 2RT5. The lower left corner is the structure of the SPOC domain of RBM15, which is displayed as a surface, and the PDB ID is 7Z27. The lower right corner is the structure of the SPOC domain of RBM15B, which is displayed as a surface, and the structure is predicted by AlphaFold. (C) Structure alignment of the SPOC domain of RBM15, RBM15B, and SPOC domain of SPEN with SMRT (YETLpSDpSL). Pink represents the SPOC domain of the SPEN, the gray represents SMRT (YETLpSDpSL), the blue represents the SPOC domain of RBM15, and the dark yellow represents the SPOC domain of RBM15B.

The domain composition of RBM15 is shown in Figure 5A. RBM15 belongs to the SPEN family, and its N-terminus contains three RNA recognition motifs (RRMs), which are important for RNA binding [89]. The C-terminus contains a Spen paralogue orthologue C-terminal (SPOC) domain, which is important for regulating interactions with other proteins, such as Setdb1 [90]. The SPOC domain is a protein domain of 15–20 kDa that is found in yeast to mammals [91]. The SPOC domain is a β-barrel structure composed of seven β-strands and several α-helices [ 92‒ 95]. There are six human proteins containing SPOC domains, namely, SPEN, RBM15, RBM15B, PHF3, and DIDO. Among them, SPEN, RBM15, and RBM15B belong to a class of proteins, including several RRM domains at the N-terminus and an SPOC domain at the C-terminus [96]. Proteins containing SPOC domains are involved in various biological processes, such as transcriptional regulation, differentiation and development [97]. Studies have shown that the SPOC domain of the SPEN protein is critical for the inhibitory effect of SPEN on the Notch signaling pathway [93]. SPEN exerts its inhibitory effect by binding to the phosphorylated serine on the LSD motif at the C-terminus of SMRT/NcoR to recruit the corepressor SMRT/NcoR-HDAC1 [93]. Recently, several studies have shown that the SPOC domain of SPEN is related to gene silencing mediated by XIST during the process of X chromosome inactivation, but the mechanism is not yet clear [98]. Two other proteins of the SPEN family, RBM15 and RBM15B, affect alternative splicing and m ^6^A modification of RNA [97]. At present, the structures of the SPOC domains of PHF3, SPEN, and RBM15 and the complex structures of the SPOC domains of PHF3 and SPEN combined with phosphorylated serine peptides have been solved [ 92, 93, 95 , 96] ( Figure 5B,C). Although there is no SPOC structure of DIDO, the SPOC structure of DIDO is predicted by AlphaFold. Structural analysis revealed that these structures of SPOC are relatively conserved overall, and in the complex structure, the positively charged region on the surface of SPOC binds phosphorylated serine [96]. Therefore, it is speculated that the SPOC domain of RBM15 also binds to phosphorylated modified peptides containing similar sequences. The SPOC domain of RBM15 may bind to proteins in the MAC-MACOM complex in a similar manner and recruit m ^6^A MTases to specific sites on RNA.

### Structure of the m ^6^A MTase predicted by AlphaFold 3

**Figure 6:**
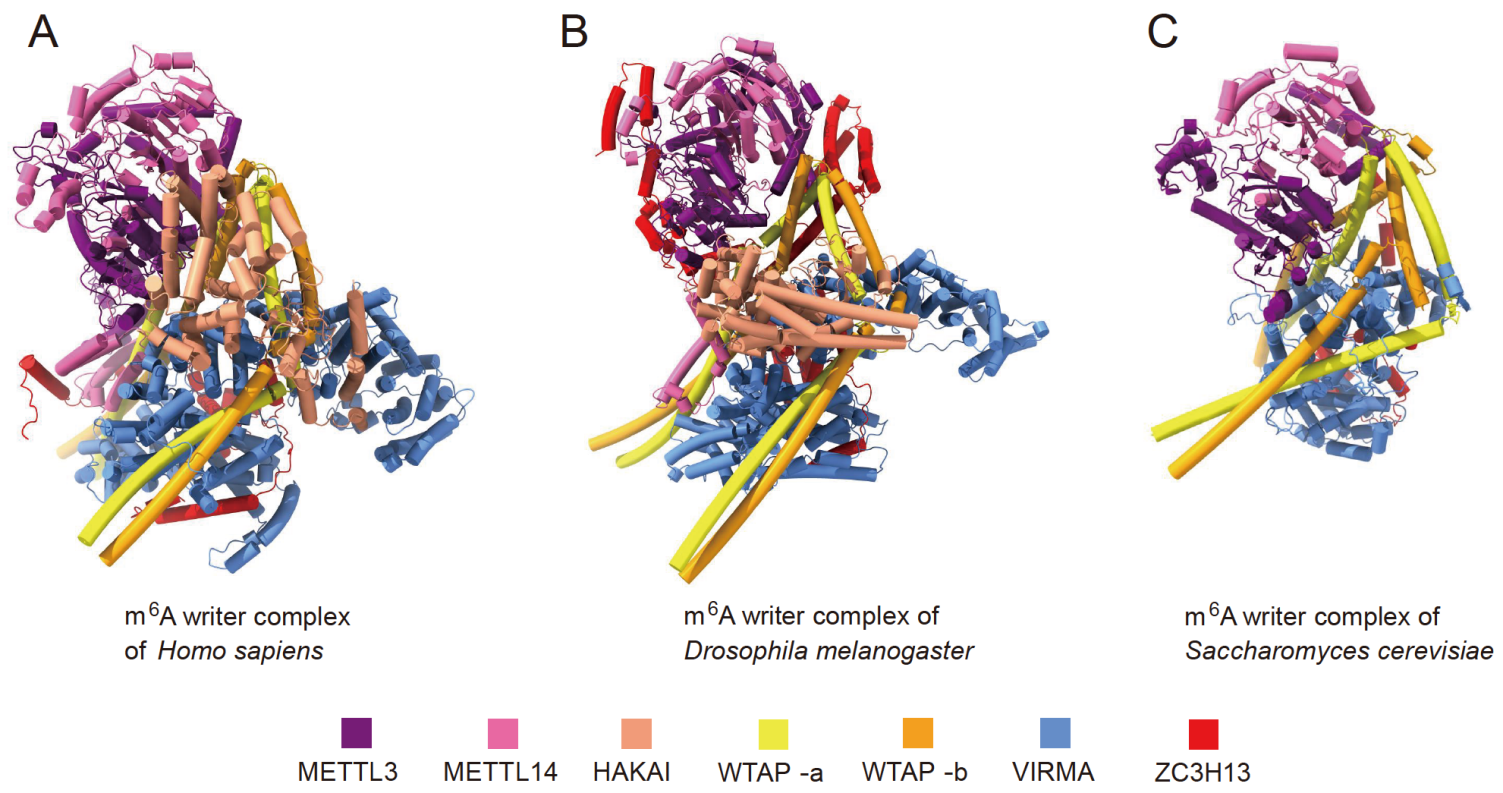
Figure 6 Structural models of m ^6^A writer complexes of *Homo sapiens* and *Drosophila melanogaster*, and *Saccharomyces cerevisiae* predicted by AlphaFold 3 (A) m6A writer complex structure of H. sapiens. (B) m6A writer complex structure of Drosophila melanogaster. (C) m 6A writer complex structure of Saccharomyces cerevisiae.

Previous studies have shown that among m ^6^A MTases, METTL3/METTL14 is highly conserved, and HAKAI/WTAP/VIRMA/ZC3H13 coevolved in metazoans and is highly conserved [63]. Our MACOM structure also shows that there are many interactions between WTAP/VIRMA/ZC3H13, and WTAP/VIRMA constitutes the rigid skeleton structure of MACOM [73]. Structural studies and interaction analysis have shown that METTL3/METTL14 mainly interact with WTAP in the MACOM complex [ 73, 85 , 99]. We also predicted the structures of the human and *Drosophila* METTL3/METTL14/HAKAI/WTAP/VIRMA/ZC3H13 and yeast METTL3/METTL14/WTAP/VIRMA/ZC3H13 complexes through AlphaFold 3. The results revealed that the structure of the m ^6^A MTase is highly conserved among these three species and that the interaction between METTL3/14 and WTAP is also conserved ( Figure 6).

## Recognition Mechanism of RNA Substrates by the MAC-MACOM Complex

A study revealed that METTL3/METTL14 specifically methylate RNA or unpaired regions of DNA [100]. In 2021, one study reported that the m ^6^A MTase is recruited to DNA regions at damaged sites exposed to ultraviolet and X-ray irradiation and that its MTase activity is required for subsequent DNA repair [101]. In the METTL3/METTL14 complex, the ZFD of METTL3 and the RGG motif of METTL14 may cause METTL3/METTL14 to bind to both RNA and DNA [99]. Among them, ZFD can specifically bind to RNA containing the 5′-GGACU-3′ motif but does not bind to RNA that does not contain the 5′-GGACU-3′ motif. However, the interaction between ZFD and specific RNAs is very weak, and the binding Kd is only a few hundred micromolar under physiological conditions [41]. However, the RGG motif in METTL14 has a strong ability to bind to substrates. After the RGG motif of METTL14 is removed, METTL3/METTL14 cannot bind to nucleic acids and will also lose MTase activity [39]. The RGG motif is a clustered sequence of arginine and glycine residues in many proteins that mediates nucleic acid‒protein or protein-protein interactions [99]. This study also revealed that the RGG motif in METTL14 directly interacts with WTAP/VIRMA. Therefore, the RGG motif of METTL14 facilitates the interaction of METTL3/METTL14 with nucleic acids and WTAP/VIRMA.

**Figure 7:**
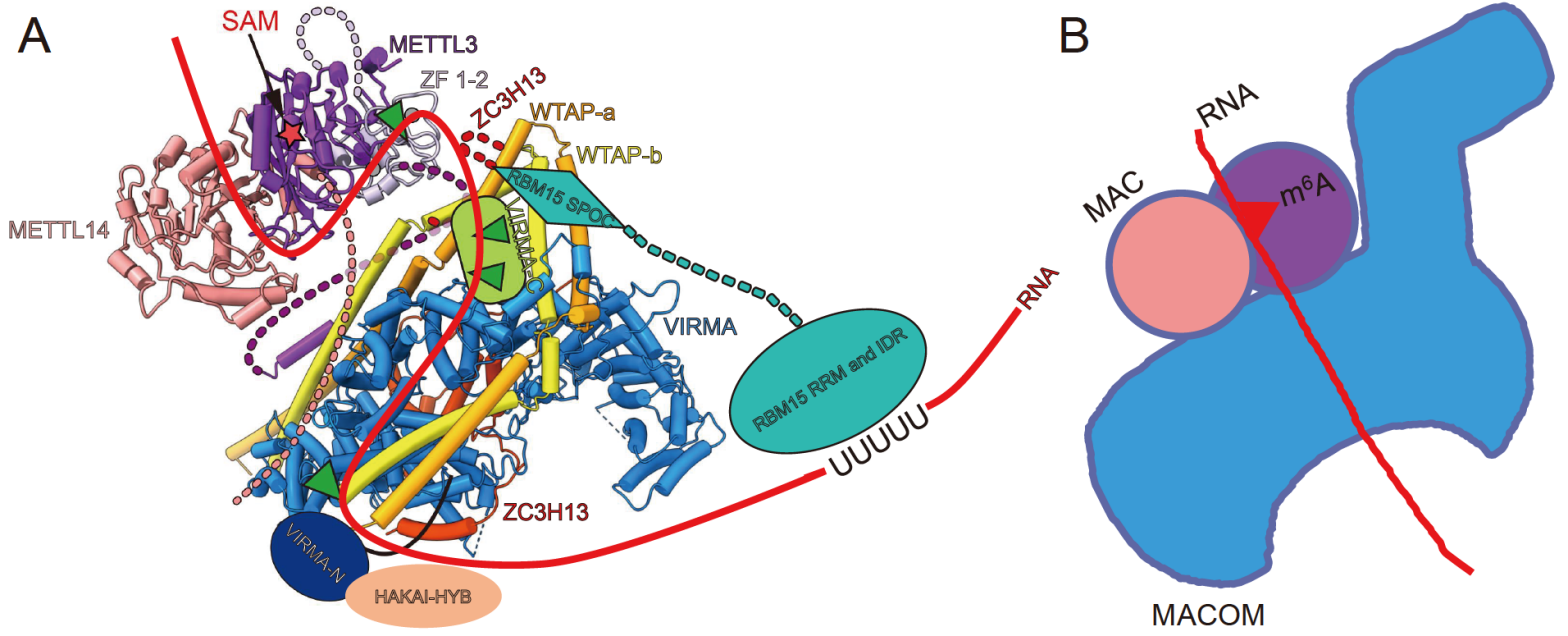
Figure 7 Potential RNA substrate binding model of the m ^6^A writer complex (A) Model of MAC-MACOM binding to RNA substrates. The red star represents the SAM. The green triangle represents the four s4U cross-linking sites, and the light coral dashed line represents the N terminus of METTL14. The purple and lavender dashed lines represent the N-terminal domain and the connection between the ZnF domain and the MTase domain of METTL3, respectively. The SUN domain and C-terminal part of VIRMA and the HYB domain of HAKAI are represented by dark blue ovals, green boxes and pink ovals, respectively. The RRM and IDR of RBM15 are represented by light blue ovals, the SPOC domain is represented by light blue diamonds, and the light blue dashed line represents the connection between the RBM15 RRM/IDR and the SPOC. The red curve represents RNA. (B) Schematic drawing of the m6A writer holocomplex with RNA, the blue horse represents the MACOM complex, the pink and peach circles represent the MAC, and the red line represents the RNA, and the red triangle represents the m6A modification site.

One study showed that WTAP/VIRMA could inhibit the binding of METTL3/METTL14 to dsDNA and maintain m ^6^A methylation activity on RNA [99]. This study revealed that METTL3/METTL14 can bind both to DNA and RNA, resulting in weak m ^6^A MTase activity of METTL3/METTL14 on RNA. However, the WTAP/VIRMA complex can reduce the binding of METTL3/METTL14 to dsDNA, thereby increasing the m ^6^A MTase activity of METTL3/METTL14 on RNA [99]. Other studies have shown that HAKAI/WTAP/VIRMA/ZC3H13 can directly bind to RNA; among them, HAKAI has the smallest impact on binding to RNA, ZC3H13 has the greatest impact on binding to RNA, and adding the HAKAI/WTAP/VIRMA/ZC3H13 complex significantly enhances its binding to RNA substrates, indicating that HAKAI/WTAP/VIRMA/ZC3H13 can directly promote the binding of m ^6^A MTase to RNA [ 73, 99, 102] ( Table 1 and Figure 7). To gain a deeper understanding of the mechanism by which WTAP-VIRMA affects the ability of METTL3/METTL14 to bind to dsDNA, the authors first investigated whether the RGG of METTL14 binds to dsDNA _50_, and the results revealed that RGGs directly bind to dsDNA _50_. In the presence of WTAP/VIRMA, the binding of RGGs to dsDNA _50_ is disrupted. The interaction between WTAP/VIRMA and METTL3/METTL14 may be stronger than that between dsDNA and METTL3/METTL14, thereby regulating the binding of METTL3/METTL14 to RNA and promoting the methylation activity of METTL3/METTL14 on RNA [99].

**Table:** **Table 1** Substrate specificities and kinetic parameters of m ^6^A RNA or DNA modifications in writers

Protein	Substrate	*K*m _(substrate)_	*K*m _(SAM)_	*K*_D_	Reference
METTL3/14	ssRNA	22	102	337.9 ± 12.1 nM	[ 99, 102]
METTL3/14	dsDNA _50_	n/a	n/a	463.5 ± 3.6 nM	[99]
METTL3/14	Human ACTB mRNA fragment	n/a	n/a	351.7 ± 4.2 nM	[73]
METTL3/14	Human MALAT1 fragment	n/a	n/a	345.1 ± 6.8 nM	[73]
METTL3/14+HAKAI/WTAP/VIRMA/ZC3H13	Human ACTB mRNA fragment	n/a	n/a	160.1 ± 3.2 nM	[73]
METTL3/14+HAKAI/WTAP/VIRMA/ZC3H13	Human MALAT1 fragment	n/a	n/a	156.9 ± 2.8 nM	[73]
HAKAI/WTAP/VIRMA/ZC3H13	Human ACTB mRNA fragment	n/a	n/a	214.0 ± 2.6 nM	[73]
HAKAI/WTAP/VIRMA/ZC3H13	Human MALAT1 fragment	n/a	n/a	274.7 ± 5.3 nM	[73]
HAKAI/WTAP/VIRMA	Human ACTB mRNA fragment	n/a	n/a	562.7 ± 76.6 nM	[73]
WTAP/VIRMA/ZC3H13	Human ACTB mRNA fragment	n/a	n/a	256.3 ± 27.9 nM	[73]
WTAP/VIRMA	Human ACTB mRNA fragment	n/a	n/a	826.3 ± 336.9 nM	[73]

In summary, the mechanism by which m ^6^A MTases recognize RNA is not fully understood. However, the current study revealed that both METTL3/METTL14 and HAKAI/WTAP/VIRMA/ZC3H13 can bind to RNA. In the METTL3/METTL14 complex, the ZnF domain of METTL3 plays a role in specifically recognizing 5′-GGACU-3′, but its ability to bind to RNA is weak, whereas the RGG in METTL14 has a strong ability to bind to nucleic acids. In the HAKAI/WTAP/VIRMA/ZC3H13 complex, all four proteins have an effect on RNA binding, among which ZC3H13 has the strongest effect, HAKAI has the smallest effect, and VIRMA can directly bind to RNA. The WTAP/VIRMA complex interacts with the RGG motif of METTL14 to regulate the binding of METTL3/METTL14 to RNA.

## Distribution of m ^6^A Modifications on RNA and Its Selective Mechanism

With the development of m ^6^A high-throughput sequencing technology, m ^6^A modification maps at the transcriptome level of various eukaryotes have been obtained, and m ^6^A modification has been shown to occur widely on eukaryotic mRNAs [ 5, 6, 103‒ 105 ]. m ^6^A modifications are concentrated mainly on mRNAs, long noncoding RNAs (lncRNAs) and tRNAs [ 106‒ 109]. In mRNAs, m ^6^A modifications are distributed mainly in the 3′ UTR, long exons and 5′ terminus [ 5, 6, 110]. Among them, m ^6^A in the 3′ UTR may be related to the stability of mRNA and mRNA transport because m ^6^A modification can affect the stability of mRNAs and their interaction with RNA-binding proteins. m ^6^A modifications in the exon region may be involved in regulating splicing and affecting the alternative splicing process of mRNAs. m ^6^A modifications near the 5′ terminus may be related to the initiation of translation and transport of mRNAs [ 5, 6, 110]. The distribution of m ^6^A modifications is also affected by the secondary structure of RNA and its interaction with RNA-binding proteins. m ^6^A accounts for approximately 0.15%–0.6% of total adenine [ 44, 111‒ 116 ], and there are approximately three m ^6^A modification sites per transcript [3]. These m ^6^A modification sites are concentrated mainly in the DRACH (D: G/A/U, R: G/A, H: U/A/C) motif [ 53, 117, 118], and approximately 5% of DRACHs contain m ^6^A modifications [5].

**Figure 8:**
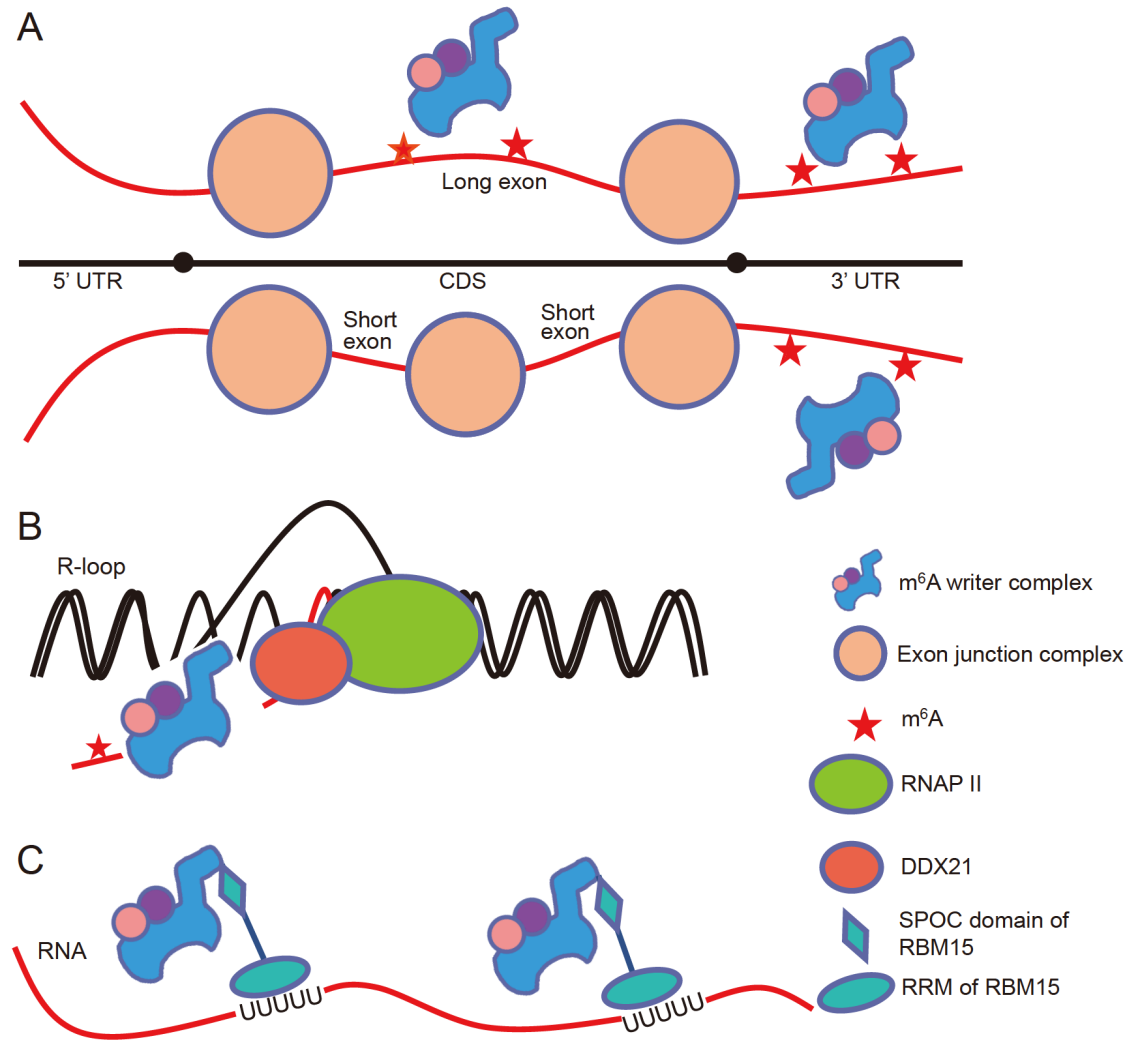
Figure 8 Model of m ^6^A selective modification (A) Model of how the exon junction complex participates in selective m6A modification. (B) Model of DDX21 and RNAP II participation in selective m6A modification. (C) Model of RBM15 participation in m6A modification.

In early sequencing studies, m ^6^A was detected mainly in the 3′ UTR, and later studies revealed that m ^6^A modifications on mRNAs are concentrated mainly in the 3′ UTR and long exons [ 5, 6, 110]. Although selective m ^6^A modification plays a critical role in m ^6^A-mediated gene regulation, the mechanism of this selectivity is still poorly understood. Notably, most studies on the regulation of m ^6^A modification selectivity have focused on mechanisms that selectively activate m ^6^A in specific regions. These activated mechanisms include the recruitment of MTases via transcription factors, RNA-binding proteins, and histone modification factors to modify nearby RNA sequences. However, known pathways, such as long internal exons and near stop codons, cannot explain the enrichment of m ^6^A in specific regions of the transcriptome, so the primary mechanisms that control m ^6^A modifications remain unknown. In 2023, He e *t al*. [119] discovered that the exon splicing complexes (EJCs) near the exon boundary act as m ^6^A suppressors, regulating the selectivity of m ^6^A modification sites on mRNAs, which determines the overall m ^6^A distribution specificity. Exon architecture affects mRNA stability and regulates gene expression through this new mechanism. At the evolutionary level, exon-intron architecture regulates mRNA stability in a m ^6^A-dependent manner in vertebrates [119]. Further studies have shown that mRNA splicing is important for the suppression of m ^6^A modification [119]. Splicing of exons with an average length of 100–200 nt inhibits m ^6^A modification, whereas splicing of abnormally long exons or terminal exons does not inhibit m ^6^A modification. What mechanism leads to this splicing- and exon length-dependent inhibition of m ^6^A methylation? Researchers have reported that EJCs inhibit m ^6^A modification near exon junctions; in the transcriptome, thousands to tens of thousands of m ^6^A sites are inhibited by bound EJCs, resulting in the special distribution of m ^6^A on the transcriptome, including enrichment in long exons, the last exon, and near stop codons. In contrast to previous studies, this study revealed that many m ^6^A modifications occur during the splicing process or after the completion of splicing. EJC depletion leads to m ^6^A hypermethylation on mRNAs, which in turn results in the recruitment of more m ^6^A reader YTHDF2, causing transcript instability [119]. Therefore, EJCs protect RNA from m ^6^A hypermethylation, which is important for maintaining normal gene expression ( Figure 8A).

In addition to the inhibitory effect of EJCs on m ^6^A modification, several other factors may be involved in regulating the site selectivity of m ^6^A modification, such as RBM15, whose RRM is involved in recognizing RNA, and the SPOC domain can recruit the MAC-MACOM complex, thereby recruiting m ^6^A MTase to specific regions on RNA and methylating nearby sites [57] ( Figure 8B). Recently, several studies have shown that R-loops, the helicase DDX21 and the m ^6^A MTase METTL3 have a coordinated effect, which plays a role in promoting transcriptional termination and maintaining genome stability [120]. Coimmunoprecipitation experiments revealed that DDX21 is a new factor that interacts with the m ^6^A MTase. Moreover, this study revealed that the R-loop, DDX21 and METTL3 clearly colocalize in the genome. When the R-loop or DDX21 is deleted, METTL3 localization to chromatin is significantly reduced, resulting in reduced m ^6^A levels on chromatin-associated RNA (caRNA), especially at transcription termination sites. Studies have shown that the ability of DDX21 to promote m ^6^A modification depends on its helicase activity. The R-loop-DDX21-METTL3 interaction jointly regulates m ^6^A modification during cotranscription and coordinates transcriptional termination and genome stability [120] ( Figure 8C). In addition to RBM15 and DDX21, other factors may be involved in regulating the binding of m ^6^A MTase to RNA and the selectivity of m ^6^A modification sites by interacting directly or indirectly with the MAC-MACOM complex.

## Conclusion

m ^6^A modifications on RNA are highly conserved in most eukaryotes. m ^6^A modification of RNA is regulated by a variety of factors, which jointly regulate m ^6^A modification activity, substrate selectivity and m ^6^A modification site selectivity. METTL3/METTL14 are two MT-A70 family proteins that form the catalytic core of the m ^6^A MTase and are highly conserved from yeast to humans. In addition to the catalytic core component MAC, more MACOM components have been found in higher animals and plants, which indicates that m ^6^A modification requires more regulation in higher organisms. Although not all organisms contain MACOM, WTAP is present in most eukaryotes, and WTAP coevolves with the METTL3/METTL14 complex. VIRMA, ZC3H13 and HAKAI coexist with WTAP in metazoans. In plants, only VIRMA, HAKAI and WTAP are in the same complex, and there may be other proteins that play similar roles as ZC3H13 in plants. Structural studies of the human m ^6^A MTase have shown that ZC3H13 uses its C-terminal binding domain to interact with VIRMA and induce conformational changes in MACOM. *In vitro* biochemical experiments revealed that ZC3H13 clearly enhances RNA binding and m ^6^A MTase activity. However, the currently analyzed MACOM complex contains only the 1492--1643 part of ZC3H13. It is difficult to analyze the molecular mechanism of ZC3H13’s role from a structural perspective. Research should further analyze the structure of the full-length *ZC3H13* gene and explore the molecular mechanism by which ZC3H13 binds to RNA and promotes m ^6^A modification. METTL3, the catalytic core component of MAC, interacts with MACOM mainly through WTAP. Although there is no structure of HAKAI in the currently analyzed MACOM structure, biochemical experiments have shown that HAKAI can interact with WTAP, VIRMA, and ZC3H13 in MACOM, HAKAI can interact with the N-terminal SUN domain of VIRMA in *Drosophila* and humans [77], and the N-terminal SUN domains of VIRMA and HAKAI were not observed in the previous structure, indicating that HAKAI may be flexible in MACOM composed of HAKAI, WTAP, VIRMA, and ZC3H13. Several studies have shown that HAKAI is very important for the stability of MACOM in *Drosophila* [80]. Therefore, the mechanism of HAKAI in MACOM needs to be further studied in the future.

m ^6^A modification has been shown to participate in a variety of biological processes by changing the stability, conformation, and folding of RNA or by directly regulating the interaction of modified RNA with binding proteins that affect RNA fate and function. Although several studies have solved the high-resolution structures of the MTD of METTL3/METTL14 and the ZFD of METTL3 in MAC, the structure of MACOM contains WTAP, VIRMA and ZC3H13, as do the medium-resolution cryo-EM density maps containing MAC-MACOM. *In vitro* RNA substrate binding and m ^6^A MTase activity assays revealed that MACOM (HWVZ and WVZ) can directly bind to RNA substrates and significantly increase m ^6^A MTase activity. Studies have shown that WTAP-VIRMA can competitively bind to METTL3/METTL14 with nucleic acids, reducing the ability of METTL3/METTL14 to bind to DNA and promoting the m ^6^A MTase activity of METTL3/METTL14 on RNA. Studies have shown that EJC, RBM15 and DDX21 can regulate the selectivity of m ^6^A modification. However, the specific molecular mechanism of the interaction between MAC and MACOM in m ^6^A MTases, the mechanism by which MAC-MACOM recognizes RNA substrates, and the mechanism by which MACOM regulates the m ^6^A MTase activity of MAC remain unclear. The complex structure of MAC-MACOM combined with RNA sheds light on which components are involved in the binding of nucleic acids and how the complex distinguishes RNA and DNA as substrates. The molecular mechanism by which the m ^6^A MTase participates in the regulation of m ^6^A modification activity and modification site selectivity remains to be revealed.
